# Cyclic RGD functionalized PLGA nanoparticles loaded with noncovalent complex of indocyanine green with urokinase for synergistic thrombolysis

**DOI:** 10.3389/fbioe.2022.945531

**Published:** 2022-08-10

**Authors:** Sha Zhang, Jinjie Li, Jiefeng Ren, Zaiyao Xue, Xinlian Qi, Quanjin Si

**Affiliations:** ^1^ Department of Geriatric Cardiology, Second Medical Center of Chinese PLA General Hospital, National Clinical Research Center for Geriatric Diseases, Beijing, China; ^2^ Medical School of Chinese PLA, Beijing, China; ^3^ Centre of Sport Nutrition and Health, Zhengzhou University, Zhengzhou, China; ^4^ Department of the Third Health Care, Second Medical Center of Chinese PLA General Hospital, National Clinical Research Center for Geriatric Diseases, Beijing, China

**Keywords:** phototherapy, PLGA nanoparticles, thrombus, plasminogen activators, targeted delivery, activated platelet

## Abstract

Thrombotic diseases have the characteristics of long latency period, rapid onset, and high mortality rate, which seriously threaten people’s life and health. The aim of this research is to fabricate a novel indocyanine green complex of urokinase (ICG@uPA) and employ the amphiphilic PEG-PLGA polymer to deliver the complex as an enzyme-phototherapeutic synergistic thrombolysis platform. The noncovalent indocyanine green (ICG) complex of urokinase (ICG@uPA) was prepared *via* supramolecular self-assembly and then encapsulated into cRGD decorated polymeric nanoparticles (cRGD-ICG-uPA NPs) by double-emulsion solvent evaporation method. Then the nanoparticles (NPs) were characterized in terms of particle size, optical properties, *in vitro* release, etc. The targeting and thrombolytic effect of the nanoparticles were studied both *in vitro* and *in vivo*. ICG@uPA and cRGD-ICG-uPA NPs displayed significantly higher photostability and laser energy conversion efficiency than free ICG. Concomitantly, the NPs exhibited selective binding affinity to the activated platelets and specific accumulation in the mouse mesenteric vessel thrombus. Significant thrombolysis was achieved *in vivo* by photo-assisted synergistic therapy with reduced dose and systemic bleeding risk of uPA. Our results prove that the functional PLGA nanoparticle loaded with the ICG@uPA offers a novel option for effective and safe thrombolytic treatment.

## Introduction

Vascular embolism disease caused by thrombosis, such as ischemic stroke and myocardial infarction, can lead to severe tissue damage and organ failure, which has become a significant cause of death and disability around the world (Benjamin et al., 2017). The early diagnosis and treatment of thrombotic diseases are of great significance to improving patient survival and preventing further development of thrombus (Landman et al., 2011; Meissner et al., 2012; Vaidya et al., 2012). As there are hardly any apparent symptoms in the early stage of thrombosis, it is difficult to detect and treat the lesions timely with the available treatments (Luo et al., 2018). Therefore, developing new strategies for thrombotic diseases is of great research interest.

Thrombolytic therapy is one of the most efficient and recommended anti-thrombus methods in clinic practice. Urokinase (uPA) is one of the commonly choices due to its antigenicity/pyrogenicity-free property, wide scope of application and affordable cost (Kunamneni et al., 2008). However, severe drawbacks of systemic bleeding, poor stability, and short half-life *in vivo* have limited its application (Matsuo, 2005; Vaidya et al., 2012; Engelmann and Massberg, 2013). With the rapid development of nanotechnology, nanomedicines have been used as drug delivery systems (DDSs) for thrombolytic agents because of their high drug-loading capacity and beneficial physical properties (Kwon et al., 2018; Huang et al., 2019; Chen et al., 2020). For instance, liposomes (Refaat et al., 2021), polymeric nanoparticles (Tan et al., 2012; Shahdeo et al., 2021), and magnetic nanoparticles (Prilepskii et al., 2018) have been used to deliver uPA to the effect of significantly increasing the *in vivo* half-life and therapeutic efficacies. Given the easily modifiable properties of the nanocarriers, pathological changes at the thrombus site such as the activated platelets (Huang et al., 2019; Li et al., 2019; Bai et al., 2020), fibrin (Kang et al., 2017; Lu et al., 2019), or thrombin (Xia et al., 2018) are often utilized as the specific targets for nanocarriers to enhance the local concentration of thrombolytic agents. Among these reported target spots, GPIIb-IIIa (αIIbβ3), a glycoprotein receptor on the activated platelet membrane, is generated during the initiation of pathological thrombotic process. GPIIb-IIIa has been found to make an important contribution to the formation and development process of thrombus by modulating the adhesion protein molecules, thus making it possible to design receptor binding nanocarriers. The peptide with cyclic arginine-glycine-aspartate structures (cyclic Arg-Gly-Asp, cRGD), which has been demonstrated to have enhanced binding affinity towards GPIIb-IIIa, is a potential candidate for thrombus specific drug delivery (Huang et al., 2008; Cui et al., 2017; Bai et al., 2020).

Phototherapy, a non-invasive therapy often including photothermal therapy (PTT) and photodynamic therapy (PDT), has been shown to induce thrombus ablation through heat and reactive oxygen species (ROS) generated by the stimulation of photosensitizers under site-specific irradiation (Singh et al., 2016; Zhang et al., 2019). Nanoparticles designed with optical properties offers minimal invasive treatment for assisting thrombolysis. For instance, gold nanoparticles have been shown to produce ROS that, when excited by visible light, leads to large voids, collapse of the blood clot, and thermally induced lysis of fibrin thrombi in mice (Walker and Zaleski, 2014; Yang et al., 2018). A recent study has reported that polypyrrole nanoparticle loaded with heparin achieved synergistic thrombolysis through precise hyperthermia with NIR laser irradiation (Lu et al., 2021). Most of the current studies packed both the thrombolytic agents and photosensitizers in a formulation that simultaneously targeted thrombus region. However, this simple combination approach faces problems of different release speed of payloads, uncontrolled co-encapsulation efficiency and uncertain stability of encapsulated agents.

Herein, a pre-coupling of the photosensitizer and thrombolytic agent before encapsulation, we supposed, would be an effective strategy that may overcome the obstacles of simple co-delivery systems and be applied for high efficacious synergistic thrombolysis. In this study, we fabricated a complex with easily adjustable photosensitizer and urokinase ratio and encapsulated the complex into targeting nanoparticles for thrombolysis studies. Indocyanine green (ICG), an NIR fluorescence dye approved by FDA with ideal optical penetration depth in tissues (Wang et al., 2018), was selected as the model photosensitizer since it exhibited strong interactions with urokinase in our pre-simulation *via* molecular docking assay. The noncovalent ICG complex of uPA (ICG@uPA) was prepared through self-assembly and the complex was further encapsulated in a PLGA nanoscale scaffold with cRGD decoration by double emulsion method. The ICG@uPA were tested for both enzyme activity and photostability since ICG has a poor photostability both in the solution and under irradiation (Kirchherr et al., 2009). A remarkable *in vivo* thrombolysis efficacy from the combination of thrombolytic therapy and phototherapy was confirmed in mice mesenteric vascular thrombus. These results demonstrated that the photo-assisted therapy based on the functionalized PLGA nanocarrier could act as a highly efficacious nanomedicine for thrombus treatment.

## Materials and methods

### Materials

Poly (lactic-co-glycolic)-poly (ethylene glycol) methyl ether (PLGA-mPEG) (PLGA: latide:glycolide 50:50, MW 5,600–8,400 Da; PEG: MW 2,000 Da) and Poly (lactic-co-glycolic)-poly (ethylene glycol)-N-hydroxysuccinimide (PLGA-PEG-NHS) (latide:glycolide 50:50, MW 5,600–8,400 Da; PEG: MW 2,000 Da) were purchased from Chongqing Yusi Medicine Co., Ltd. Cyclic RGD (cyclo (L-Arg-Gly-L-Asp-D-Phe-L-Lys) peptide was purchased from Hangzhou GuTuo Biochem Co., Ltd, and the urokinase (uPA) for injection (100,000 U) was purchased from Wuhan Humanwell Pharmaceutical Co., Ltd. Indocyanine green (ICG) was obtained from Xi’an Ruixi Biological Technology Co., Ltd. 1,3-Diphenylisobenzofuran (DPBF) was purchased from Sigma-Aldrich. N, N-Dimethylformamide (DMF), poly (vinyl alcohol) (PVA) (MW ≈ 47,000 Da), dichloromethane (DCM), acetone, triethylamine (TEA) and rhodamine 6G were purchased from Shanghai Macklin Co., Ltd.

### Preparation of ICG@uPA and peptide functionalized polymer

Molecular docking studies were firstly performed by Autodock 4.2 software to predict the complex’s structure model and the affinity between ICG and uPA. The three-dimensional (3D) structures of urokinase were obtained from the PubChem Database, and the crystal structure of ICG was obtained from the RCSB Protein Database Bank. The binding energy was calculated using AutodockTools 1.5.6. All 3D structures were visualized by Pymol 2.5.

Based on the intermolecular interaction between ICG and uPA, the noncovalent ICG complex of uPA (ICG@uPA) was fabricated *via* self-assembly in aqueous solution. Specifically, freshly prepared ICG aqueous solution (1 mg/ml) and uPA aqueous solution (45,000 U/ml) were mixed with a molar ratio of 10:1. After 2 h stirring, the mixture was transferred into a dialysis bag (MWCO = 3,500 Da) and further dialyzed for 48 h with frequent water changes to remove unreacted ICG. The coupling percentage of ICG was determined by UV-Visible spectrophotometer. The enzyme activity of ICG@uPA was determined with an urokinase activity ELISA kit (Mlbio, Shanghai, China). All of the above experiments were carried out in the dark, and the purified ICG@uPA was collected and freeze-dried for further use.

The cRGD peptide was conjugated to PLGA-PEG-NHS *via* the nucleophilic substitution reaction (Li et al., 2017). Firstly, PLGA-PEG-NHS and cRGD were both dissolved in DMF and mixed well (1:1 M ratio), then pH of the mixture was regulated to near 8.0 by TEA and further stirred at 4°C overnight. The resulting solution was subsequently dialyzed against deionized water for 48 h (MWCO = 3,500 Da) to remove the reagent and unreacted cRGD. Final product of PLGA-PEG-cRGD was characterized by ^1^H NMR (Bruker, Germany) in DMSO-*d6* and FTIR spectrometer (Nicolet, United States).

### Preparation of cRGD-ICG-uPA NPs

The ICG@uPA loaded nanoparticles with cRGD decoration (cRGD-ICG-uPA NPs) were prepared through the double-emulsion solvent evaporation method (Raza et al., 2021). Firstly, the aqueous solution of ICG@uPA was added into the organic solution of PLGA-PEG-cRGD (in DCM/acetone 3:2, v/v) at the volume ratio of 1:3. The mixture was then sonicated with the ultrasonic homogenizer (JY92-IIN, SCIENTZ, China) for 3 min (50W, 3 s on/off cycle) in ice bath. After the formation of primary emulsion (water in oil, W/O), it was mixed with double volume of 2% PVA aqueous solution and further emulsified (30 W, 3 s on/off cycle) for another 2 min to produce the double-emulsion solvent (water in oil in water, W/O/W). The resulting emulsion was rotarily evaporated (Yamato RE212-B, Japan) until the solution became transparent. The obtained solution was centrifuged at 12,000 rpm for 10 min and the precipitates were rinsed for three times to remove unloaded ICG@uPA and residual reagents. Finally, the cRGD-ICG-uPA NPs were dispersed in saline and stored at 4°C for subsequent use. The unloaded nanoparticles (UNPs) were prepared by the same method, except that ICG@uPA in the first step was substituted by double-distilled water. The non-modification nanoparticles (ICG-uPA NPs) were constructed using the cRGD modification-free polymer (PLGA-mPEG) by the same method.

### Characterizations of the NPs

The absorption spectra of ICG@uPA, cRGD-ICG-uPA NPs and unloaded nanoparticles were examined firstly by UV-Visible spectrophotometer. The photobleaching assay was performed by NIR irradiation to evaluate whether ICG@uPA and the NPs could enhance the photostability of ICG. Specifically, freshly prepared cRGD-ICG-uPA NPs, ICG@uPA, and ICG aqueous solution were respectively exposed to the laser irradiation of 1 W/cm^2^, and their absorption intensity at 780 nm were recorded over time. The morphology of cRGD-ICG-uPA NPs was investigated by transmission electronic microscope (TEM; JEM1200EX, JEOL, Japan) after negatively stained. Besides, hydrodynamic diameter, polydispersity index (PDI) and zeta potential of the NPs were determined at pH 7.4 in PBS by dynamic light scattering (DLS; Nano series ZEN 3600, Malvern, United Kingdom). The encapsulation efficiency (EE) and drug-loading capacity (LC) were determined indirectly by measuring the unloaded ICG@uPA in the supernatant according to previous reports (Zhang et al., 2016; López-Machado et al., 2021). Specifically, the supernatant during centrifuge and rinsing was collected and diluted to a fixed volume, then the amount of un-loaded ICG@uPA was measured by UV-Visible spectrophotometer. The EE and LC of obtained cRGD-ICG-uPA NPs were calculated as per the following equations

$$EE\left(\text{\%}\right)=\frac{amount\;of\;feeding\;ICG@uPA\;-\;amount\;of\;un‐loaded\;ICG@uPA}{amount\;of\;ICG@uPA\;in\;feeding}\;\times 100\text{\%,}$$ (1)

$$LC\left(\%\right)=\frac{mass\;of\;feeding\;ICG@uPA-mass\;of\;un‐loaded\;ICG@uPA\;}{total\;mass\;of\;the\;encapsulated\;nanoparticles}\times 100\text{\%}\text{.}$$ (2)

As the cRGD-ICG-uPA NPs were administrated by injection, its stability was investigated by determining the hydrodynamic diameter of the NPs after 5-fold–1000-fold dilution by PBS and after dispersion in PBS containing 10% FBS (fetal bovine serum) at 37°C for 1 week. Besides, the NPs were dispersed in PBS and stored at 4°C for 15 days, then the average size and drug loading of ICG@uPA were determined to evaluate the storage stability of cRGD-ICG-uPA NPs.

### Determination of laser-induced thermogenesis and singlet oxygen production

Excited photosensitizers can convert the laser energy to heat and generate reactive oxygen species (ROS) through its reaction with oxygen, consequently triggering the thrombus ablation. To investigate the phototherapeutic potential of cRGD-ICG-uPA NPs, both the photothermal and photodynamic effect were individually studied through the thermogenesis and singlet oxygen production assay. The thermogenesis effect was studied by detecting the temperature variation of the samples. Specifically, cRGD-ICG-uPA NPs aqueous dispersions of different ICG concentrations (2.5, 5, and 10 μg/ml) were prepared and placed into the cuvette. The assay was carried out at 37°C on a constant temperature magnetic stirrer (C-MAG HS7 control, IKA, Germany), the laser source was offered by an 808 nm power-tunable infrared laser (Laserwave, Beijing, China) and the temperature fluctuation of the sample solutions was measured over time with a laser intensity of 1 W/cm^2^. The temperature elevation curves of ICG@uPA and free ICG solutions were also examined by the same method. As for singlet oxygen production assay, the experiments were divided into three groups: free ICG solution, ICG@uPA solution and cRGD-ICG-uPA nanoparticles solution, with the same concentration of ICG (5 μg/ml). Then 1,3-diphenylisobenzofuran (DPBF) was applied as the detection probe since its specific absorption peak would be quenched in the presence of singlet oxygen. In the light of reference, DPBF was dissolved in EtOH (0.1 mM) as probe stock solution, the stock solution was then added to an equal volume of the prepared cRGD-ICG-uPA NPs solution prior to the assay (Entradas et al., 2020). The amount of singlet oxygen generated during the irradiation was quantitatively evaluated through the DPBF absorption value reduction curves. Additionally, the enzyme activity before and after the irradiation (1 W/cm^2^ for 12 min) was determined by the ELISA kit to validate the effect of high temperature and singlet oxygen on the activity of urokinase during the phototherapy.

### *In vitro* release studies

The centrifugation method was used to investigate the *in vitro* release pattern of ICG@uPA from cRGD-ICG-uPA NPs. Specifically, prepared NPs were dispersed into PBS (pH = 7.4), followed by incubation at 37°C in a thermostatic oscillation incubator. The incubation solution was sampled at every predetermined time point and supplied with the same volume of fresh PBS. Then samples were centrifuged by 12,000 rpm and the supernatant was collected to measure the released ICG@uPA. To further examine whether synchronous release was achieved, cumulative released of ICG@uPA from cRGD-ICG-uPA NPs was determined by both the uPA and ICG according to the following equation.

$$Cumulative\;release\left(\%\right)=\frac{mass\;of\;released\;ICG/uPA}{mass\;of\;ICG/uPA\;encapsulated}\times 100\text{\%}\text{.}$$ (3)

Therein, the released ICG was determined by UV-Visible spectrophotometer and released protein was calculated by Bradford protein assay kit (Beyotime, China).

### Binding affinities to activated platelets

In order to study the targeting ability of the NPs with or without cRGD modification, binding affinities of the NPs to activated platelets was investigated *via* flow cytometry analysis according to the previous report (Huang et al., 2008). Fresh blood samples were collected from eyeballs of the BALB/c mice anesthetized by pentobarbital sodium (50 mg/kg). Mice were then sacrificed under anesthesia *via* cervical dislocation after the blood collection. The fresh quiescent platelets were prepared carefully by gradient centrifuging of the whole blood. The activated platelets were obtained by incubating the freshly prepared platelets with thrombin buffer at room temperature for 10 min. Then PBS, ICG-uPA NPs (20,000 U/ml), and cRGD-ICG-uPA NPs (20,000 U/ml) were put into the quiescent or activated platelets individually. The reaction was performed in the presence of 5 mM calcium chloride for 30 min. The platelets were subsequently centrifuged and rinsed repeatedly, and then analyzed with the BD Accuri C6 flow cytometer (BD Biosciences, United States).

### *In vitro* thrombolysis assay

The *in vitro* thrombolysis assay was evaluated in a transparent bottle containing 10 ml of sample solutions. The experiments were divided into five groups: saline, saline with NIR illumination (illumination group), free urokinase solution, targeted nanoparticle solution and targeted nanoparticle solution with illumination (combination group) (*n* = 3 per group). Specifically, fresh mouse blood was collected as previously mentioned and aliquoted into several tubes to form natural clot. Then saline, free urokinase solution (20,000 U/ml) and cRGD-ICG-uPA NPs solution (20,000 U/ml of uPA) were separately put in the bottles. NIR laser irradiation (1 W/cm^2^) was subsequently applied to both the illumination group and the combination group, with the irradiation time of 10 min for three times. All of the thrombi were weighed before and after lysis, and the following formula was used to evaluate thrombi lysis.

$$Thrombi\;lysis\left(\%\right)=\frac{the\;loss\;weight\;of\;thrombi}{the\;initial\;thrombi\;weight}\times 100\text{\%}\text{.}$$ (4)

### Development of the mouse mesenteric thrombus model and *in vivo* thrombus targeting assay

All animal experimental procedures were approved and performed following the principles of the Life Sciences Institutional Review Board of Zhengzhou University. Male BALB/c mice aged 5–6 weeks old were purchased from Henan SKB Laboratory Animal Co. (Henan, China) and acclimatized for a minimum of 7 days on standard feed with free access to water. Since ferric chloride-induced thrombus is a well-established animal thrombosis model used in the evaluation of novel antithrombotic drugs, the ferric chloride-induced mouse mesenteric artery thrombosis model was developed in our study based on previous research (Bonnard and Hagemeyer, 2015; Singh et al., 2016). Specifically, mice were anesthetized with pentobarbital sodium (50 mg/kg) and the hair of each mouse was removed to avoid its interference in fluorescence. The mesentery of mice was carefully spread out through a small incision and the suitable vessel was chosen for thrombosis. Then a filter paper with the width of 1 mm was soaked with 5% (w/v) FeCl_3_ and applied on the vessel for 5 min. Finally, the filter paper was removed, and the vessel was flushed with saline. In order to confirm the thrombosis, each mouse was intravenously administrated with 50 μl of rhodamine 6G solution (1 mg/ml) to label the platelets and leukocytes. The treated vessel was observed under an inverted fluorescence microscopy. For *in vivo* thrombus targeting assay, after the application of FeCl_3_ and flushed with saline, the incision was carefully sutured and cRGD-ICG-uPA NPs (20 U/g) of 100 μl were then administered through tail vein injection. Whole-body scan of the mice was subsequently performed by a fluorescence imaging system (Biolight Biotechnology Co., Ltd., China) (excitation wavelength: 730 nm; emission wavelength: 800 nm).

### Thrombolysis studies on mice mesenteric vascular thrombosis models

*In vivo* thrombolysis effect of the NPs was performed on an inverted fluorescence microscopy (ECLIPSE Ti, Nikon, Japan). The mice were randomly divided into five groups: the saline, NIR laser irradiation (illumination group), cRGD-ICG-uPA NPs (100 U/g) with or without irradiation, and free urokinase (300 U/g), with five mice in each group. After the establishment of mesenteric vascular thrombosis model and the administration with rhodamine 6G as previously described, the mice were placed in a petri dish and put on the heating pad to keep their body temperature constant. Then 100 μl of sample solutions were administrated through tail vein for each group. The irradiation was applied after 15 min of injection and the laser intensity was set at 0.3 W/cm^2^. The total irradiation time was set at 10 min with interval, and images of the mesenteric vessel were recorded every 10 min. To analyze the thrombolytic effect of each group, thrombus size variation was assayed by the area of TRITC fluorescent signals using ImageJ 1.53K software.

### Bleeding risk and biosafety study

Systemic thrombolysis is usually associated with significant risk of bleeding. The bleeding risk of cRGD-ICG-uPA NPs was evaluated by the tail bleeding assay and coagulation function assay on healthy BALB/c mice. The mice were randomly arranged into four groups: the saline, free uPA solution (300 U/g), and cRGD-ICG-uPA NPs solution (300 U/g) with or without irradiation, with five mice in each group. Then 100 μl of sample solutions were administered individually through tail vein every other day for three times in total. The mice in the illumination group and combination group were provided with interval NIR laser irradiation (0.3 W/cm^2^) for 10 min after each injection, and the clinical signs were recorded during the study for all mice. After the last administration and irradiation, the mice were anesthetized for the tail bleeding assay. Briefly, a distal 5-mm segment of the tail was amputated, and the tail was immediately immersed into a 50 ml tube containing saline at 37°C, then the bleeding time of each mouse was recorded. At the end of the experiment, the mice were sacrificed, and blood samples were collected to assay the plasma coagulation factors by an automatic coagulation analyzer (Rayto Life and Analytical Sciences Co., Ltd., China). Besides, the major organs including heart, liver, spleen, lung, kidney of each mouse were harvested and fixed by 4% paraformaldehyde for histological analysis.

### Statistical analysis

Statistical analysis was performed using SPSS 21 software, and the statistical significance was assessed through the student’s *t* test or one-way ANOVA test. Data were presented as mean ± SD, with *p* values of <0.05 indicating statistical significance.

## Results and discussion

### Characterization of ICG@uPA and NPs

**FIGURE 1:**
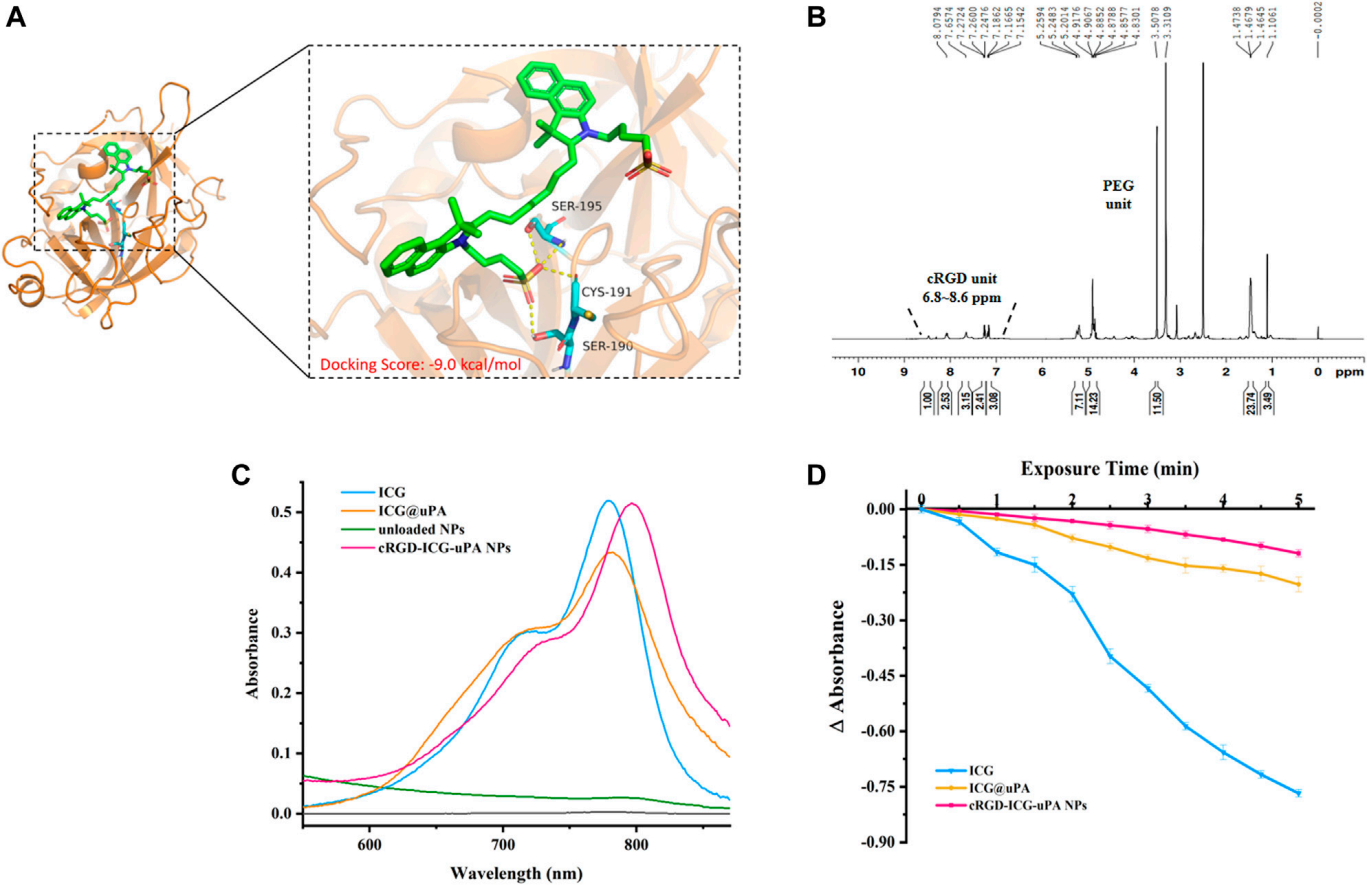
**(A)** The predicted binding mode between ICG and uPA. The key residues of ICG interacting with uPA are shown in the enlarged view. **(B)** H^1^ NMR spectrum of cRGD-PEG-PLGA (600 MHz, DMSO-*d6*, 300 K). **(C)** Absorbance spectra of free ICG, ICG@uPA, unloaded NPs, and cRGD-ICG-uPA NPs in aqueous solutions. **(D)** Absorbance reduction curves of free ICG, ICG@uPA and cRGD-ICG-uPA NPs in aqueous solution under irradiation with 808 nm laser (1 W/cm^2^).

By using molecular docking, we found that ICG could act on uPA with a low binding energy (−9.0 kcal/mol) (Figure 1A), suggesting that ICG and uPA had significant interactions. The coupling percentage of the ICG was about 32% as measured by UV-Vis spectroscopy, and ELISA assay revealed that uPA complex of ICG caused no obvious reduction in enzymatic activity (more than 80% of the enzyme activity was retained). H^1^ NMR (Figure 1B) and FTIR (Supplementary Figure S1) demonstrated the conjugation of cRGD on PLGA-PEG frame. As expected, the characteristic peak of ICG appeared in absorbance spectra of ICG@uPA and cRGD-ICG-uPA NPs, and a red-shift of the absorption peak of ICG@uPA was observed after it was encapsulated into the NPs (Figure 1C). The EE and LC of ICG@uPA was about 62 and 3.8%, respectively.

Photobleaching experiment exhibited that about 92% free ICG was degraded under irradiation with NIR laser for 5 min, while ICG@uPA and the NPs showed only approximately 24 and 14% degradation of absorbance respectively (Figure 1D), indicating that ICG@uPA helped to prevent the photobleaching of ICG and the photostability was an outstanding feature of cRGD-ICG-uPA NPs as phototherapeutic agents.

**FIGURE 2:**
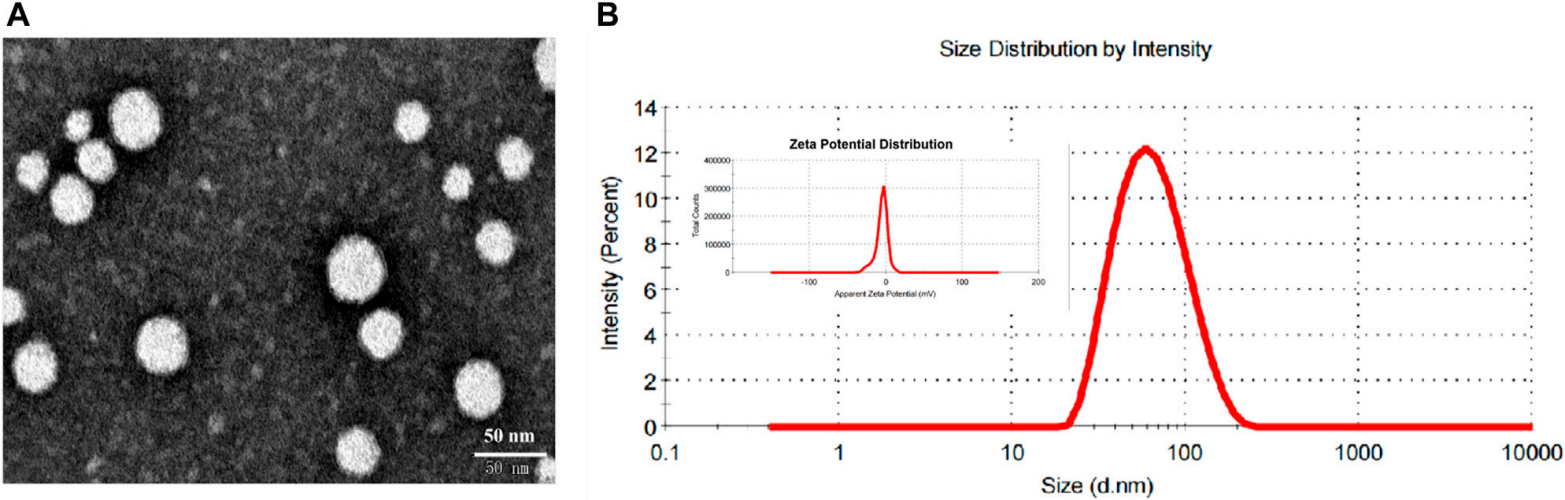
**(A)** Representative TEM image of cRGD-ICG-uPA NPs. **(B)** The size distribution and zeta potential distribution of cRGD-ICG-uPA NPs measured by dynamic light scattering.

Morphology assay revealed that the cRGD-ICG-uPA NPs were dispensed evenly with spherical morphology and relatively homogeneous particle size (Figure 2A). The cRGD-ICG-uPA NPs exhibited an average particle diameter of 68 ± 2 nm (PDI of 0.17 ± 0.01) and a zeta potential of −5.0 ± 0.3 mV (Figure 2B). The larger particle size observed in DLS test than TEM test could be attributed to the dehydration of the NPs during morphology assay. Additionally, the average hydrodynamic diameter of unloaded nanoparticles and non-modification nanoparticles were 46 ± 4 nm (PDI of 0.20 ± 0.07) and 53 ± 1 nm (PDI of 0.15 ± 0.01), with zeta potential of −15.2 ± 0.3 mV and −9.7 ± 0.6 mV, respectively. The particle size of cRGD-ICG-uPA NPs increased with ICG@uPA encapsulation and cRGD modification. And the charge of cRGD-ICG-uPA NPs was higher than that of the unloaded nanoparticles and untargeted nanocarriers, this could be explained by that the isoelectric point of both cRGD peptide (9.49) and urokinase (8.4–8.7) is greater than the pH (7.4) of the PBS solution. The overall negative surface charge of cRGD-ICG-uPA NPs may help prevent the nanospheres from aggregating and being adsorbed onto serum proteins according to previous report (Aggarwal et al., 2009). Stability studies showed that there was no significant change in the hydrodynamic diameter of the cRGD-ICG-uPA NPs after up to 1000-fold dilution and after dispersion in 10% FBS/PBS for 1 week (Supplementary Figure S2), which may be attributed to the repulsion between the NPs and the shielding effect of the superficial PEG chain. After 15 days’ storage at 4°C, the average size of the NPs remained stable (Supplementary Table S1) and the contents of ICG and uPA were determined as 91.2 and 95.0% of the initial values separately. In a word, the PEGylated nanocarriers showed desirable physio-chemical properties of *in vitro* characterization.

### Laser-induced thermogenesis and singlet oxygen production of NPs

**FIGURE 3:**
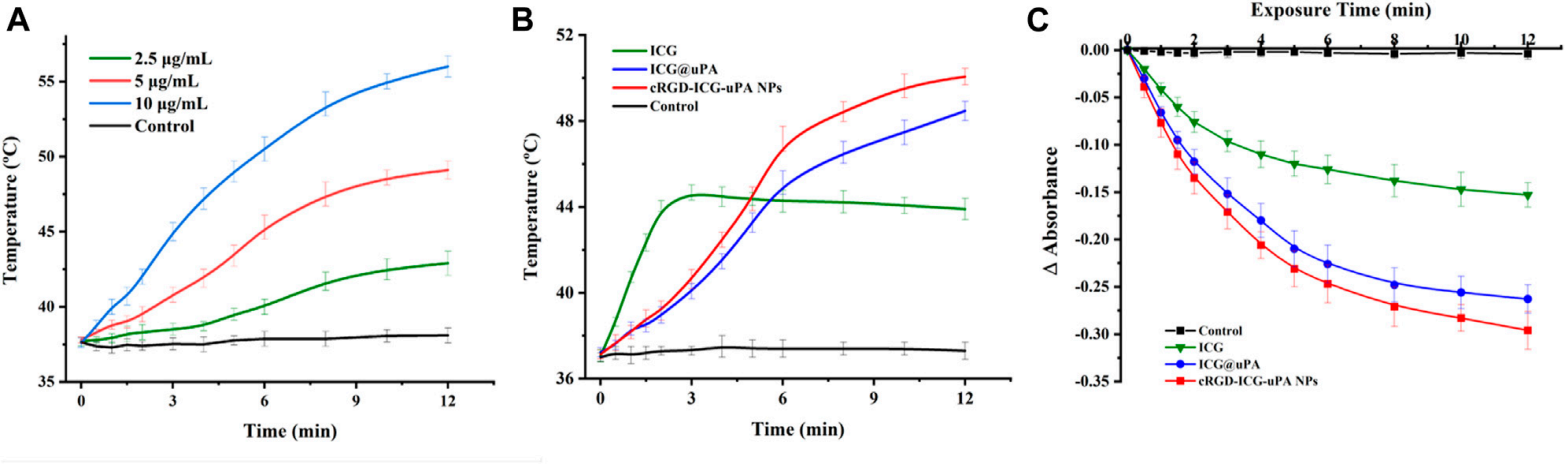
**(A)** Temperature elevation curves of cRGD-ICG-uPA NPs aqueous solutions of different ICG concentration (2.5, 5, and 10 μg/ml) under NIR irradiation (1 W/cm^2^) (*n* = 3). **(B)** Temperature elevation curves of free ICG, ICG@uPA, and cRGD-ICG-uPA NPs in aqueous solutions under NIR irradiation (1 W/cm^2^) (*n* = 3). **(C)** Singlet oxygen yield of free ICG, ICG@uPA, and cRGD-ICG-uPA NPs exposed to NIR laser irradiation (1 W/cm^2^) in aqueous solutions (*n* = 3).

As the key factor in phototherapy, the photosensitizer, whether or not it could efficiently convert light energy, is essential to the phototherapeutic outcomes. Indocyanine green (ICG), an ideal candidate, has been used in clinical fluorescence imaging for its large molar absorption coefficient, high fluorescence quantum yield, non-toxicity and deep penetration into tissue layers. Thermogenesis assay was firstly carried out to evaluate the phototherapeutic potential of cRGD-ICG-uPA nanoparticles. As shown in Figure 3A, under irradiation with 808 nm laser, the temperature of cRGD-ICG-uPA NPs aqueous solution increased fast and showed close relationship with the concentration of ICG, while there was no temperature change in control group. Following irradiation for 10 min, the temperature of 5 μg/ml sample rose to approximately 48°C and reached 55°C at the concentration of 10 μg/ml solution. Studies have shown that high-temperature exposure poses thermal damage to surrounding healthy tissues and potentially induces local inflammation due to thermal diffusion, and mild-temperature (42–45°C) photothermal treatment has been proven to achieve ideal therapeutic performance (Huang et al., 2020; Xiao et al., 2020; Yi et al., 2021). Accordingly, the concentration of ICG was set at 5 μg/ml in our study. The temperature elevation assay exhibited that ICG@uPA and the NPs had longer elevation time than the free ICG solution at the same concentration of 5 μg/ml (Figure 3B), suggesting the constructed nanoparticles could support a more sustained and stable photothermal therapy.

Then singlet oxygen determination was conducted *via* molecular quenching method. As shown in Figure 3C, a continuous reduction of the absorbance of DPBF was observed in all groups during first 6 min with obviously higher singlet oxygen yields of ICG@uPA and the NPs. Owing to photodegradation of free ICG, the absorbance reduction of ICG group slowed down post 6 min, whereas the other two groups kept a relative constant reduction trend. In accordance with thermogenesis results, this results also clearly showed that ICG complex of uPA could maintain higher photostability than free ICG under laser irradiation. After 12 min, singlet oxygen yield of ICG@uPA and cRGD-ICG-uPA NPs reached nearly twice that of the free ICG group. The enzyme activity assay showed that the enzyme activity of urokinase remained 85.6 ± 3.2% after the irradiation. In short, these results indicated that cRGD-ICG-uPA NPs could effectively enhance the thermogenesis and singlet oxygen production of free ICG with remained urokinase activity under irradiation, which can serve as a potential candidate for enzyme-phototherapeutic thrombolysis.

Attempts have been made to encapsulate ICG into nanoparticles, which are generally beneficial for prolonging the *in vivo* circulation time, but the improvement for molecule stability is still limited and usually needs complex fabrication processes (Zhu et al., 2018). In this study, we used supramolecular self-assembly to create a simple noncovalent complex of ICG@uPA with enhanced stability under NIR laser exposure and in solutions. After being loaded into the complex and nanoparticles, the contact between ICG and surrounding water molecules is reduced, and the interaction between ICG molecules is also weakened, which is conducive to reducing the photobleaching of ICG. Besides, the dispersion effect helps prevent further aggregation between the ICG molecules and improves the problems of aggregation induced quenching of single ICG solution. At the same time, the structure of ICG@uPA determines that the ICG can achieve appropriate energy absorption, matching and transfer, and ensure the stability of ICG in the system, as well as a significant improvement in light energy conversion efficiency.

### *In vitro* release assay

**FIGURE 4:**
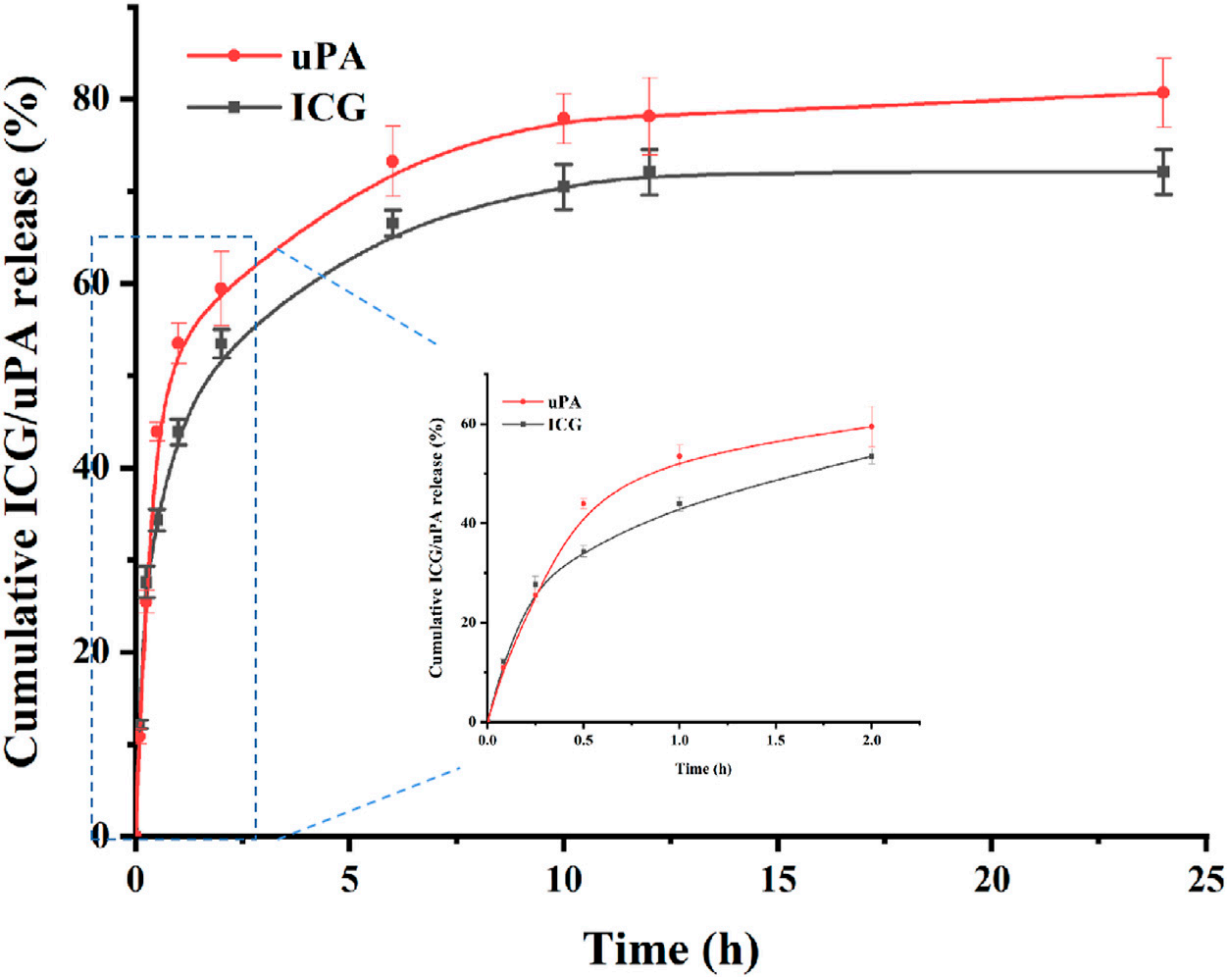
The release profile of uPA and ICG from cRGD-ICG-uPA NPs in PBS, pH 7.4 (*n* = 3).

In order to simulate the drug release and thrombolysis process *in vivo*, *in vitro* release and thrombolysis assays were carried out. The release of ICG@uPA from nanoparticles was determined by both the ICG and urokinase. As displayed in Figure 4, the cumulative release curve showed that the release trends of uPA and ICG from the nanoparticles were basically similar, which confirmed that there was stable binding between urokinase and ICG. Accordingly, ICG@uPA had a relative fast release phase from cRGD-ICG-uPA NPs during the first 1 h and then most of ICG@uPA was released in 10 h. Slow release was observed up to 24 h with the release ratio of approximately 80% till the end of the test. According to *in vivo* thrombus targeting assay (Figure 7), the NPs were found to accumulate into thrombus at 10 min post-injection, and gradually gathered in 30 min in mouse mesenteric vascular thrombosis models, which meant that most of the ICG@uPA could be retained for its therapeutic efficacy before the NPs gathered around the thrombus. According to the American Stroke Association and clinical thrombolysis guidelines, the first 4.5 h following an ischemic stroke are considered “the golden period” for getting thrombolytic therapy, and the impact of treatment is time-dependent within the therapeutic time-window (Appelros and Terént, 2015; Powers et al., 2018). The rapid drug release of ICG@uPA from the NPs within the first 1 h could provide adequate concentration of ICG and urokinase for therapeutic concentration to eliminate the thrombus timely, then prolonged drug release during later stage may also help retain durable thrombolytic effects.

### Binding affinities to activated platelets

**FIGURE 5:**
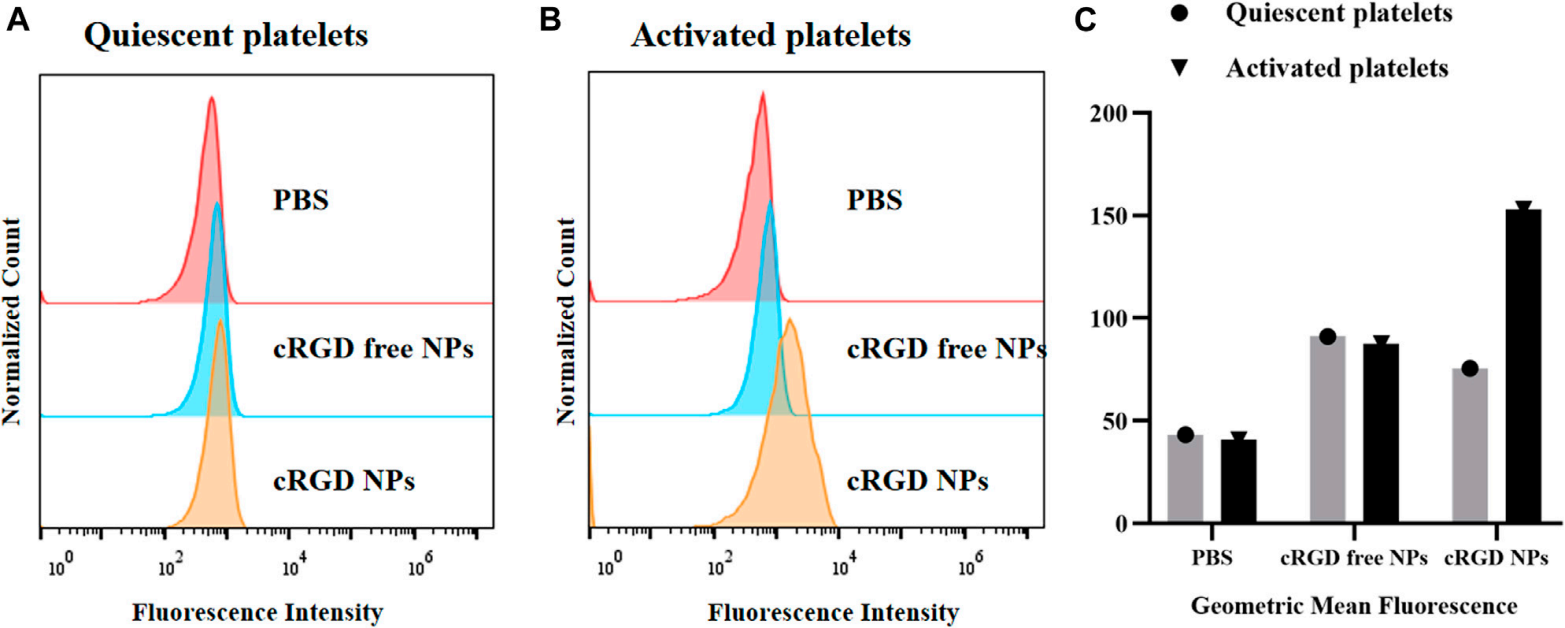
Flow cytometry analysis of binding affinity of the NPs to **(A)** freshly prepared quiescent platelets and **(B)** activated platelets treated with thrombin. **(C)** The geometric mean fluorescence of PBS, ICG-uPA NPs, and cRGD-ICG-uPA NPs treated platelets.

Based on the potential effects on inducing site-specific thrombolysis of cRGD-ICG-uPA NPs, we examined whether the nanocarriers could bind to activated platelets specifically. The binding assay was analyzed on both the nanoparticles with/without cRGD decoration by flow cytometry. As displayed in Figure 5, after incubation with quiescent platelets, the fluorescence intensity of PBS group was lower than that of the other two groups, suggesting there was partly nonspecific binding between the NPs and platelets. Comparatively, after incubation with activated platelets, the cRGD-ICG-uPA NPs showed significant increase of ICG-associated fluorescence (Figure 5), while cRGD free nanoparticles showed no obvious change than those treated with quiescent platelets. The cRGD-mediated enhanced binding rate indicated that cRGD-ICG-uPA NPs could bind to activated platelets of thrombus specifically and therefore, achieve specific accumulation in the thrombus region.

### *In vitro* thrombolysis assay

**FIGURE 6:**
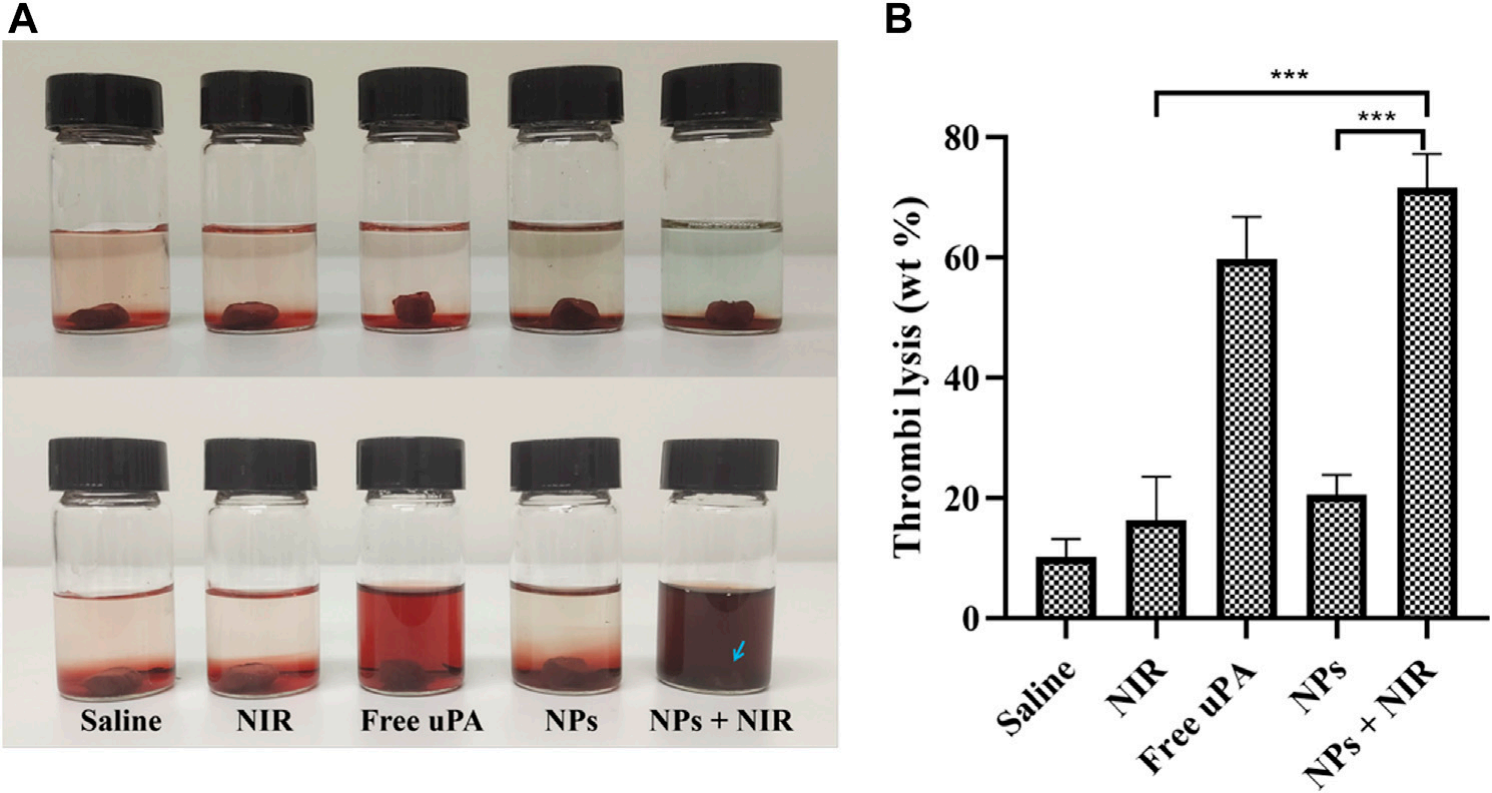
**(A)** Representative photos of thrombus treated with saline, saline with NIR irradiation (1 W/cm^2^), free uPA (20,000 U/ml), cRGD-ICG-uPA NPs (20,000 U/ml), and cRGD-ICG-uPA NPs combined with NIR laser irradiation (20,000 U/ml; 1 W/cm^2^). **(B)** The weight loss of thrombus in corresponding groups of *in vitro* thrombolysis test after 4 h (*n* = 3). Data were represented as mean ± standard deviation (SD) ***p < 0.001.

The *in vitro* thrombolysis assay exhibited expected significant thrombus ablation with a thrombolysis rate of 72% (Figures 6A,B) in the combination group, indicating that our thrombolytic strategy of combining urokinase with phototherapy was feasible. Besides, the urokinase group also displayed effective thrombus ablation with the thrombolysis rate of 60%, which may be attributed to the local high concentration and close action between urokinase and thrombi in the bottle. The average weight of blood clots in the NPs group lost 21% of their initial weight in 4 h. The unapparent thrombolytic effect of single NPs *in vitro* may be due to the slow release of ICG@uPA under static conditions. On the other hand, the suppressed thrombolytic activity of the NPs may also help prevent systemic hemorrhage before binding to the thrombus.

Most reported co-loaded nanoparticles have difficulty controlling the synergistic release of two or more payloads, which has a great impact on the outcomes of synergistic therapies (Teuschl et al., 2017; Zhu et al., 2018). Herein, the PLGA nanoparticles loaded with preformed ICG@uPA realized adjustable proportion of components and synchronous release of the payloads, which meant that the NPs could serve as a suitable platform for tunable and synergistic thrombolysis *via* the administration of *ex vivo* laser. The cRGD-ICG-uPA NPs was proven to generate reactive oxygen species and hyperthermia upon light irradiation, further inducing augmented thrombolysis *in vitro*. One of the possible mechanisms is that phototherapy helps with the disintegration of the thrombus, thus enhancing the contact between urokinase and thrombus. On the other hand, urokinase induced fibrinolysis could also help the phototherapeutic thrombolysis by accelerating the disintegration of thrombus skeleton.

### *In vivo* thrombus targeting study

**FIGURE 7:**
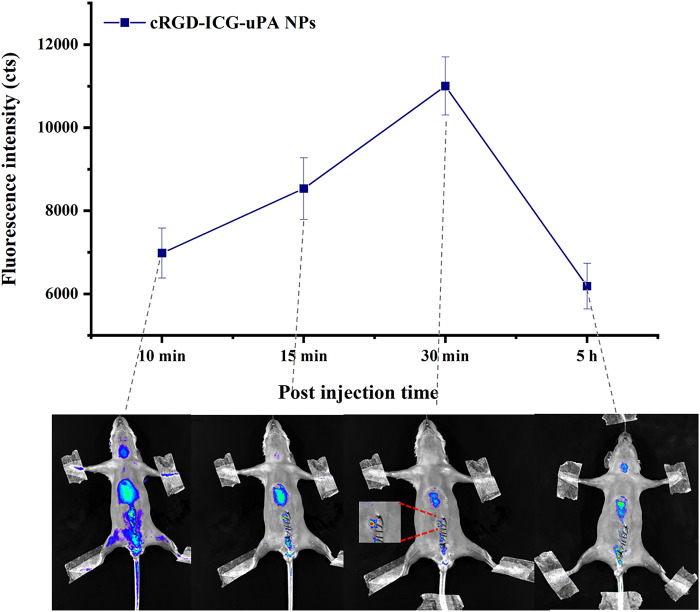
The average fluorescence intensity of thrombus after intravenous injection of cRGD-ICG-uPA NPs (20 U/g) on mouse mesenteric vascular thrombosis models, and *in vivo* fluorescence images at representative time points (*n* = 3).

To investigate the accumulation of cRGD-ICG-uPA NPs in thrombus, *in vivo* thrombus targeting study was further carried out since ICG fluorescence imaging has been proven to be a relatively simple and informative technique for NIR bioimaging (Alander et al., 2012; Bai et al., 2020). The successful establishment of thrombosis model was confirmed by fluorescent microscopy as previously described. Then *in vivo* thrombus targeting ability of the NPs was studied. As shown in Figure 7, cRGD-ICG-uPA NPs were found to accumulate into thrombus at 15 min post-injection, and gradually gathered in 30 min. Afterwards, the intensity of the whole-body fluorescence gradually decreased over time. Relative weak signals were detected at 5 h post administration, and no fluorescence signal was detected at 24 h. The results suggested that cRGD-ICG-uPA NPs could accumulate in thrombus quickly after administration. Accordingly, the irradiation time point for *in vivo* thrombolysis studies was set at 15 min post administration to activate the ICG at a relative high concentration. The result demonstrated that cRGD-ICG-uPA NPs could accumulate into the thrombus as expected and gradually be excreted, which contributed to the enhancement of the thrombolysis efficacy and reduction of the systemic bleeding.

### Thrombolysis studies on mice mesenteric vascular thrombosis models

**FIGURE 8:**
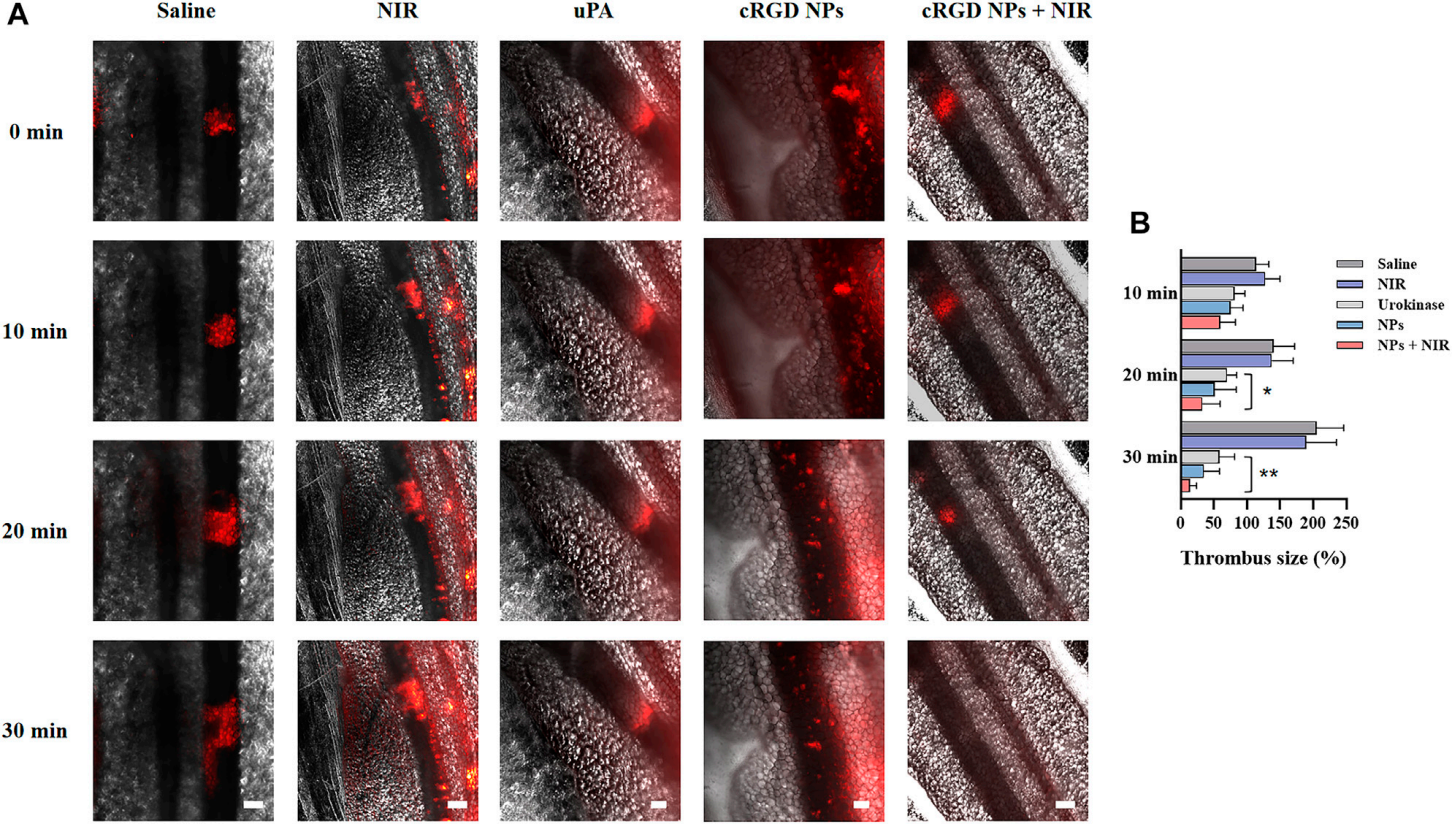
**(A)** Representative fluorescence images of mice mesenteric thrombus treated with saline, NIR laser irradiation (0.3 W/cm^2^), free uPA (300 U/g), cRGD-ICG-uPA NPs (100 U/g), and cRGD-ICG-uPA NPs combined with NIR irradiation (100 U/g; 0.3 W/cm^2^). The images were taken at time points of 0, 10, 20 and 30 min post the formation of thrombus (scale bar: 100 μm). **(B)** The thrombus size variation in corresponding groups of *in vivo* thrombolysis study (*n* = 5). Data were represented as mean ± SD *p < 0.05, **p < 0.01.

Based on the above findings, *in vivo* thrombolysis study was evaluated by the size changes of thrombus on mouse mesenteric thrombosis models. As displayed in Figure 8, the size of thrombus expanded quickly in the saline and NIR irradiation groups since their formation, and their average size nearly doubled in 30 min. The thrombus of the free uPA group was reduced to about 58% of the original size in 30 min, whereas the thrombus in the cRGD-ICG-uPA NPs group exhibited about 65% ablation with one third of uPA dosage. Since free uPA would be rapidly eliminated, it is difficult to achieve satisfactory *in vivo* thrombolysis efficacy through one-time administration. After the addition of fixed-point irradiation of 808 nm laser, the thrombi treated with cRGD-ICG-uPA NPs significantly shrank to approximately 14% of the original size in 30 min. This phenomenon may be explained by the heat and reactive oxygen species produced during the phototherapeutic process, which has been proven to disintegrate fibrin skeleton structure and other thrombus components (Walker and Zaleski, 2014; Lu et al., 2021). It is noteworthy that thrombus-localizing phototherapy not only causes hyperthermal and ROS induced thrombolysis, but also makes it easier for antithrombotic medications to penetrate deep into the thrombus, resulting in a phototherapeutic-amplified thrombolysis action. These results exhibited favorable *in vivo* thrombolysis efficacy of cRGD-ICG-uPA NPs with one third of free uPA dosage. Besides, with the addition of 808 nm laser irradiation, thrombolytic process was effectively accelerated. In this way, the synergistic thrombolysis strategy could realize precise and high efficacious thrombolytic efficacy.

### Bleeding risk and biosafety study

**FIGURE 9:**
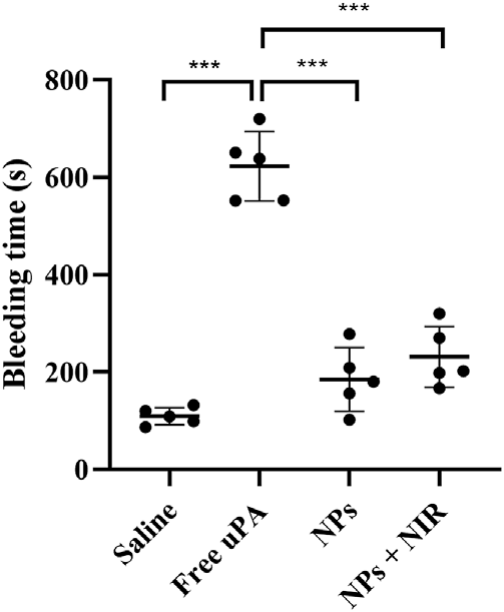
Tail bleeding time of mice injected with saline, free uPA (300 U/g), cRGD-ICG-uPA NPs (300 U/g), and cRGD-ICG-uPA NPs with NIR laser irradiation (300 U/g; 0.3 W/cm^2^) (*n* = 5). Data were represented as mean ± SD ***p < 0.001.

Bleeding risk and coagulation system reaction are the main obstacles restricting the clinical application of traditional plasminogen activators. As shown in Figure 9, the average hemostasis time in free uPA group was up to about 600 s, whereas the single cRGD-ICG-uPA NPs and combination groups had a much shorter hemostasis time of approximately 200 s. This obvious reduced hemostasis time indicated that systemic bleeding effect of uPA may be significantly reduced after it was encapsulated into cRGD-ICG-uPA NPs. In accordance with hemostasis time, the results of plasma coagulation factor assay (Table 1) exhibited prolonged prothrombin time (PT) and activated partial and thromboplastin time (APTT) of the free uPA group in comparison to the control group. An obvious decrease in fibrinogen content (FIB) was also observed, while only slightly prolonged APTT was observed in cRGD-ICG-uPA NPs and combination groups. These results suggested that cRGD-ICG-uPA NPs could contribute to reducing the bleeding risk of uPA *in vivo*.

**TABLE 1:** PT, APTT, and FIB values of mice treated with saline, free uPA (300 U/g), cRGD-ICG-uPA NPs (300 U/g), and cRGD-ICG-uPA NPs (300 U/g) with NIR laser irradiation (0.3 W/cm^2^) (*n* = 5).

Group	PT^a^ (s)	APTT^b^ (s)	FIB^c^ (g/L)
Saline	10.4 ± 0.6	20.4 ± 2.0	0.8 ± 0.3
Free uPA	13.2 ± 1.3	26.8 ± 7.3	0.5 ± 0.2
cRGD-ICG-uPA NPs	10.4 ± 0.5	22.0 ± 0.7	0.7 ± 0.3
cRGD-ICG-uPA NPs + irradiation	10.9 ± 0.8	23.6 ± 3.7	0.8 ± 0.5

a PT: prothrombin time.

b APTT: activated partial thromboplastin time.

c FIB: fibrinogen content.

During the treatment period, no apparent signs of depilation, anorexia, or other animal toxicity symptoms were observed in any group. Histological study was further employed to investigate the *in vivo* toxicity of the NPs. The sections of major organs stained with H&E (Supplementary Figure S3) showed that there was no noticeable toxicity and inflammatory cells in each group. Besides, no distinct apoptosis of cells in the major organs was observed according to the TUNEL staining images of saline, cRGD-ICG-uPA NPs, and combination groups (Supplementary Figure S4). Safety concern of nano-sized drug delivery systems is a key reason that hinders their application, such as the biocompatibility and organ cumulative toxicity of the nanocarriers. In recent years, a number of nano-sized biodegradable copolymeric medicines have been developed and can be found either in clinical trials or on the market (Souery and Bishop, 2018). Biodegradable polymers have the advantages of controlling molecular weight and thoroughly degrading into nontoxic compounds before removal from the body *via* metabolic processes. We observed no obvious toxicity on the mice treated with cRGD-ICG-uPA NPs, and no abnormal cell morphology and apoptosis was found in main organs, which may be due to the biocompatible composition and metabolism properties of the NPs.

## Conclusion

In this study, we successfully synthesized the ICG complex of uPA (IGC@uPA) by a facile self-assembled method and encapsulated it into the cRGD peptide modified PLGA nanoparticles, which effectively improved the efficacy of uPA and achieved higher thrombolytic efficiency in combination with phototherapy. In addition, the thrombus targetability of cRGD-ICG-uPA NPs was confirmed by flow cytometry and *in vivo* imaging system. This assembled functional nanocarrier overcomes the limitations of short circulation time and excessive dose needed of thrombolytic agents and exhibits a synergistically enzyme-phototherapeutic anti-thrombus efficacy. Furthermore, the side effect of systematic bleeding of urokinase was significantly reduced after coupling and encapsulation. In short, the PLGA nanocarriers loaded with fabricated ICG@uPA provide a safe and effective therapy for thrombus with high spatiotemporal specificity and low bleeding risk.

## Data Availability

The original contributions presented in the study are included in the article/Supplementary Material, further inquiries can be directed to the corresponding author.
