# Supplementary material for: Cyclic RGD functionalized PLGA nanoparticles loaded with noncovalent complex of indocyanine green with urokinase for synergistic thrombolysis
Source: Front Bioeng Biotechnol. 2022 Aug 10;10:945531. doi: 10.3389/fbioe.2022.945531 (PMC9399888; doi:10.3389/fbioe.2022.945531)
Supplement: Supplementary file 1 [file DataSheet1.pdf]

Supplementary Material

Supplementary Figures

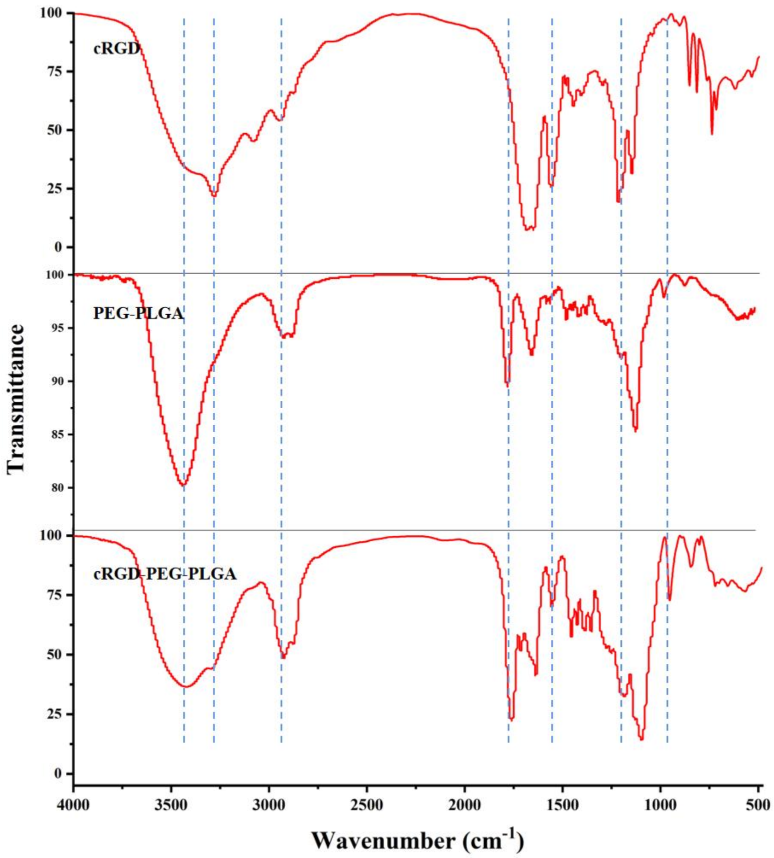

Supplementary Figure 1. The FTIR spectra of cRGD, PEG-PLGA, and cRGD-PEG-PLGA.

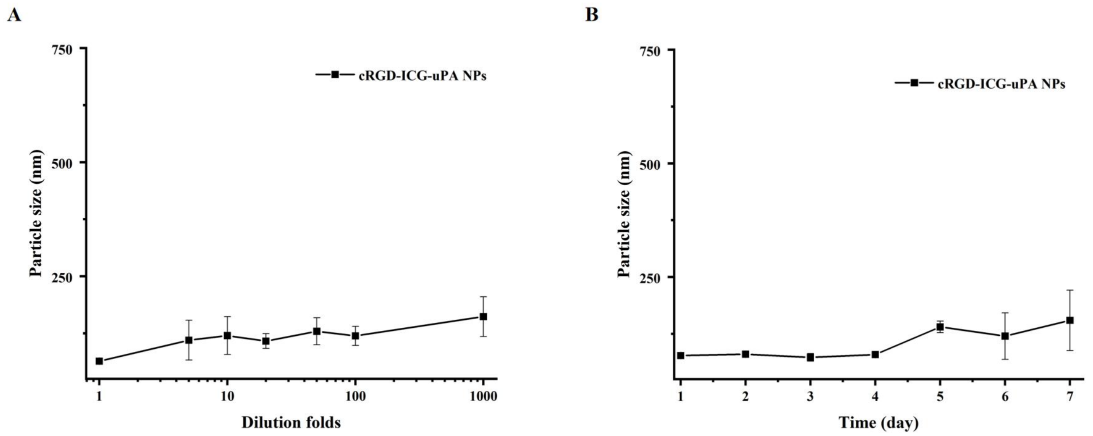

Supplementary Figure 2. The hydrodynamic size of cRGD-ICG-uPA NPs (A) diluted by different dilution factors (n=3) and (B) dispersed in PBS containing 10% FBS at 37 °C for one week (n=3).

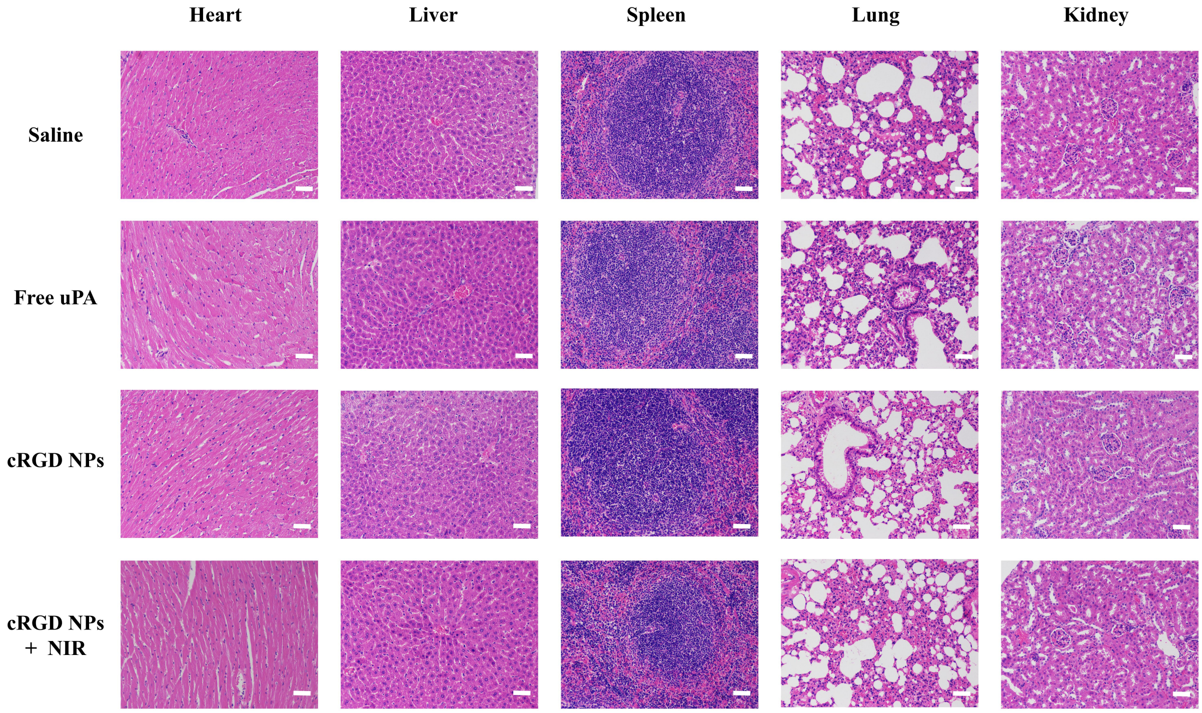

Supplementary Figure 3. Representative HE staining images of the major organs in mice treated with saline, free uPA (300 U/g), cRGD-ICG-uPA NPs (300 U/g), and cRGD-ICG-uPA NPs with NIR irradiation (300 U/g; 0.3 W/cm^2) (n = 5) (scale bar: 50 μm).

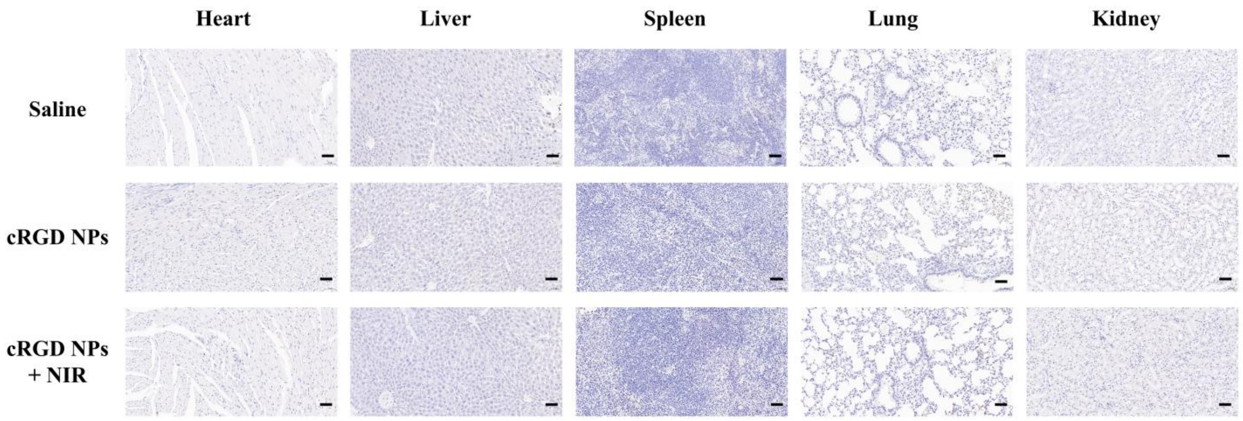

Supplementary Figure 4. Representative TUNEL staining images of the major organs on mice treated with saline, cRGD-ICG-uPA NPs (300 U/g), and cRGD-ICG-uPA NPs with NIR laser irradiation (300 U/g, 0.3 W/cm^2) (n = 5) (scale bar: 50 µm).

Supplementary Table

Supplementary Table 1. The hydrodynamic size, polydispersity index (PDI), and zeta potential of cRGD-ICG-uPA NPs on day 1 and day 15 post preparation.

cRGD-ICG-uPA NPs	Average size (nm)	Polydispersity index	Zeta potential (mV)
Day 1	68 ± 2	0.17 ± 0.01	-5.0 ± 0.3
Day 15	64 ± 2	0.16 ± 0.00	-2.7 ± 0.1
